# Role of Lipid Metabolism and Signaling in Mammalian Oocyte Maturation, Quality, and Acquisition of Competence

**DOI:** 10.3389/fcell.2021.639704

**Published:** 2021-03-05

**Authors:** Ranjha Khan, Xiaohua Jiang, Uzma Hameed, Qinghua Shi

**Affiliations:** ^1^First Affiliated Hospital of USTC, Hefei National Laboratory for Physical Sciences at Microscale, The CAS Key Laboratory of Innate Immunity and Chronic Disease, School of Basic Medical Sciences, Division of Life Sciences and Medicine, CAS Center for Excellence in Molecular Cell Science, Collaborative Innovation Center of Genetics and Development, University of Science and Technology of China, Hefei, China; ^2^Institute of Industrial Biotechnology, Government College University, Lahore, Pakistan

**Keywords:** lipid metabolism, oocyte development, oocyte maturation, fertilization, obesity

## Abstract

It has been found that the quality of oocytes from obese women has been compromised and subsequent embryos displayed arrested development. The compromised quality may be either due to the poor or rich metabolic conditions such as imbalance or excession of lipids during oocyte development. Generally, lipids are mainly stored in the form of lipid droplets and are an important source of energy metabolism. Similarly, lipids are also essential signaling molecules involved in various biological cascades of oocyte maturation, growth and oocyte competence acquisition. To understand the role of lipids in controlling the oocyte development, we have comprehensively and concisely reviewed the literature and described the role of lipid metabolism in oocyte quality and maturation. Moreover, we have also presented a simplified model of fatty acid metabolism along with its implication on determining the oocyte quality and cryopreservation for fertilization.

## Background

The female ovaries are destined for the growth and development of oocytes as well as the production of sex hormones that influence the menstrual cycle (Zhu et al., 2020). The complete cycle of oocyte maturation is a complex, continuous and tightly regulated process in mammals (Pepling and Spradling, 2001; Gunesdogan and Surani, 2016). The key step during this process is the acquisition of oocyte competence to develop as an embryo after its fertilization. Though all the factors that control the developmental competence are still not clear, however, it has been known that both the oocyte quality and competence acquisition are linked to the metabolism of oocytes (Sirard, 2011). The role of lipids in energy production, as the precursors of steroid hormones and signaling molecules suggest that the intracellular lipid content of oocytes should be carefully estimated during oocyte maturation. Furthermore, the oocyte activation is also closely linked to the mobilization of lipid reserves.

To understand the role of lipids in controlling the oocyte development, we have comprehensively reviewed the literature and described the contribution of lipid metabolism in the oocyte maturation and quality. We have also presented a simplified model of β-oxidation of fatty acid in the process of oocyte growth and maturation that will further facilitate a better understanding of lipid metabolism in controlling the oocyte quality (an oocyte’s intrinsic developmental potential) and its acquisition of competence.

## Cumulus-Oocyte Complex: The Dynamic Unit of Oocyte Metabolism

The maturation and competence acquisition of oocyte is governed by maternal signals and ovarian follicular microenvironment that are mediated by the bidirectional communication between somatic and germinal cells (Matzuk et al., 2002). Specialized granulosa cells surrounding the oocytes in antral follicles, named cumulus cells, are involved in the acquisition of oocyte developmental competence. These cells are physically and metabolically linked with an oocyte and form the cumulus-oocyte complex (COC), a chief functional and dynamic unit that is pivotal in oocyte metabolism (Tanghe et al., 2002). Since oocytes are glycolytically less active and require energy from carbohydrates, fatty acids and amino acids to achieve full maturation, these energy sources are provided to oocytes by cumulus cells from the external follicular fluid (FF) as well as from internal storage inside the COCs.

To understand the role of cumulus cells in lipid metabolism of COCs, an elaborative study was conducted on bovine cumulus cells and it was found that these cells control lipid metabolism during cytoplasmic oocyte maturation. Transmission electron microscopy displayed different lipid droplets (LDs) and membrane-bound vesicles in metaphase II denuded oocytes and cumulus enclosed oocytes (CEO). Denuded oocytes had lower lipid contents and the global transcriptomic analysis showed that various genes are differentially expressed in denuded and CEO. The local lipogenesis and lipolysis factors, such as fatty acid synthase and hormone-sensitive phospholipase proteins were detected in both denuded and oocytes groups, however, a significant reduction of fatty acid synthase protein was observed in the denuded oocytes group as compared to the CEOs indicating impaired lipogenesis in the absence of cumulus cells (Auclair et al., 2013). Hence, the removal of cumulus cells during *in vitro* maturation can affect lipid metabolism in the oocyte and can lead to suboptimal cytoplasmic maturation. Overall, cumulus cells may influence oocyte by changing the consumption of nutritive storage via regulation of local fatty acid synthesis and lipolysis to provide energy for maturation. Thus, energy metabolism balance is vital for oocyte maturation and acquisition of developmental competence.

During the past few decades, little attention has been given to the lipid or fatty acid metabolism associated with the oocyte development despite knowing the higher lipid contents in oocytes and embryos of various mammals such as pig, sheep and cows (McEvoy et al., 2000). Generally, free fatty acids in FF are transported in the form of dynamic fatty acid complex and their main function is to serve as a source of energy (Ferguson and Leese, 2006). Higher fatty acid concentration in the blood due to increased lipolysis of adipose tissue can induce lipotoxic effects on COC morphology and embryo quality (Leroy et al., 2005; Jungheim et al., 2011). Thus, balanced amount of various fatty acid concentration is required for optimal oocytes growth and development. Interestingly, higher concentration of saturated fatty acids even affects the post-fertilization developmental competence of *in vitro* matured oocytes, while monounsaturated fatty acids like oleic acid initiate the normal developmental competence. The adverse effects of saturated fatty acids can be reversed by the addition of a balanced amount of palmitic acid (C16) (Shibahara et al., 2020), stearic acid (C18) (Ranneva et al., 2020), and oleic acid during *in vitro* maturation of oocytes (Wu et al., 2010; Aardema et al., 2011). Thus, the composition as well as a balanced amount of saturated and unsaturated free fatty acids in the FF is critical for the developmental competence of oocytes. Common fatty acids constituting the composition of FF in various mammalian species have been summarized in Table 1.

**TABLE 1:** Most common fatty acids constituting lipid polymer in mammals.

Name	Carbon number	Formula	Saturation
Myristic acid	14	C_14_H_28_O_2_	Saturated
Palmitic acid	16	C_16_H_32_O_2_	Saturated
Stearic acid	18	C_18_H_36_O_2_	Saturated
Oleic acid	18	C_18_H_34_O_2_ (C-9)	Monounsaturated
Linoleic acid	18	C_18_H_32_O_2_ (C-9, 12)	Polyunsaturated
α-linoleic acid	18	C_18_H_30_O_2_ (C-9, 12, 15)	Polyunsaturated
Arachidonic acid	20	C_20_H_32_O_2_ (C-5, 8, 11,14)	Polyunsaturated
Adrenic acid	22	C_22_H_36_O_2_ (C7, 10, 13,16)	Polyunsaturated

On the other hand, *in vitro* experiments revealed that higher free fatty acid concentration in the oocyte growth medium can cause a massive increase of neutral lipids in the surroundings of COCs. The increased neutral lipid contents did not affect the developmental competence of oocytes, however, a lower blastocyst rate was observed. Subsequent *in vivo* experiments displayed that an increased level of free fatty acids can only affect cumulus cells if there is a substantial increase in the storage of triglycerides (Aardema et al., 2013). Thus, the cumulus cells of COCs can protect the maturing oocytes from an elevated level of free fatty acids by increasing the intracellular lipid storage.

## Lipid Composition and Metabolism in COC

Generally, free fatty acids, also known as non-esterified fatty acids (NEFA), are linked with serum albumin that acts as a carrier protein and helps in the transportation of these insoluble fatty acid through the circulation system (Simpson et al., 1980). These fatty acids can also be stored in the form of lipoprotein particles such as low density lipoproteins (LDLs), very low density lipoproteins (VLDLs), and high density lipoproteins (HDLs) (Gautier et al., 2010). Examination of FFs in various species revealed the presence of triacylglycerol and fatty acids. Human FF examination revealed the presence of a major amount of HDL (Valckx et al., 2012). These HDL particles are serum-derived and are the major lipoproteins present in the FFs because the follicle basement membrane is permeable only to serum proteins ≤300 KDa, thus, excluding LDL and VLDL (Jordanov and Boyadjieva-Michailova, 1974). However, human granulosa-derived lutein cells can also generate LDL and VLDL through ApoB-100 marker. Thus, the LDL and VLDL which are observed in FFs are originally produced by ovarian cells (Dunning et al., 2014b).

**FIGURE 1:**
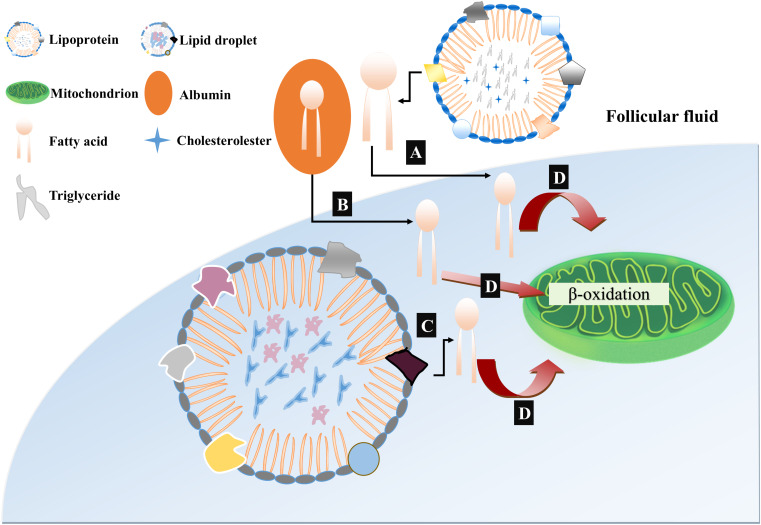
Proposed model of free fatty acid (FFA) mobilization and catabolism in COC. **(A)** Free fatty acids or non-esterified fatty acids (NEFAs) are incorporated with serum albumin and are transported to follicular fluids by fatty acid carrier or directly diffused through lipid bilayer. **(B)** The mobilization of triacylglycerol from lipoproteins in follicular fluids occurs due to enzymatic action of lipoprotein lipases through which free fatty acids are generated and become available for cellular uptake. **(C)** Intracellular triacylglycerols are stored in the cumulus cells and oocytes in the form of lipid droplets. These lipid droplets are activated and further liberate free fatty acids by lipase mediated hydrolysis. **(D)** All the liberated free fatty acids either intracellularly from lipid droplets or through transporter molecules from follicular fluids, are metabolized in the mitochondria and ATP is generated through β-oxidation.

Oocytes of different mammalian species contain different lipid droplet contents. Oocytes of pigs, cows and dogs possess a large amount of lipids and appear dark under an optical microscope while mouse and human oocytes have a substantially lower amount of lipid droplets and their cytoplasm is more transparent (Apparicio et al., 2012). These lipid contents are used by mammalian eggs to gain most of their ATP through mitochondrial oxidation. The process of early embryogenesis in mammals is dependent on β-oxidation derived ATP because early embryo is glycolytically less active (Dumollard et al., 2009). Thus, based upon the recent findings about the generation, mobilization and utilization of fatty acids during oocyte growth and development, we have presented a model of lipid metabolism providing ATP for developing oocytes and embryos to underpin the β-oxidation of fatty acids (Figure 1). To be noted, pyruvate and fatty acids are the major sources of energy supply during oocyte development, however, the balance between the generation of ATP through pyruvate or from fatty acid oxidation is completely incomprehensible. Furthermore, both proportion of ATP provided by fatty acid oxidation to developing oocyte and whether these fatty acids are obtained from endogenous lipid droplets or exogenous sources are also unclear.

Previously, coherent anti-Stokes Raman scattering (CARS) was used to determine the number, spatial distribution and chemical content of lipid droplets in mouse eggs and embryos (Bradley et al., 2016). Josephine et al. utilized CARS imaging with deuterium labeling of oleic acid to check the turnover of fatty acids within lipid droplets of living mouse eggs and found that pyruvate removal caused the increased loss of labeled oleic acid and also promoted the dispersion of lipid droplets. Subsequently, inhibition of β-oxidation leads to increased uptake of pyruvate and reduced ATP production along with clustering of lipid droplets (Tatsumi et al., 2018). Further examination revealed that there is a compensatory relationship between inhibition of pyruvate uptake and β-oxidation of fatty acids, in which both reactions caused the successive deletion of ATP (Bradley et al., 2019). Thus, β-oxidation of fatty acids and pyruvate oxidation have an equal contribution in ATP production for mouse oocyte development.

Pig farming is widely used for meat production and mostly pig embryos are produced *in vitro*. Generally, immature pig oocyte contains around 156 ng lipid contents much higher than cattle and sheep oocytes which usually have 58 and 4 ng, respectively (McEvoy et al., 2000). Interestingly, higher endogenous lipid contents has been observed in the pig oocytes and embryos which shows a dark appearance. Recent studies have been undertaken to show the potential importance and role of these higher lipid contents in pig oocytes. A comprehensive study proved that pig oocytes used endogenous triglyceride as an energy source during *in vitro* maturation and that most (91–97%) of the ATP produced during embryo development comes from oxidative phosphorylation, half of which come from β-oxidation of NEFA (Sturmey and Leese, 2003). Altogether, these studies demonstrated that NEFA is the essential source of ATP production during the mammalian oocyte maturation and development.

In contrary to that NEFA also play an important role in the energy metabolism of the developing oocytes in various mammalian species, some studies have controversial results about the exact role of lipid metabolism. Higher concentration of NEFA also caused metabolic stress and jeopardized oocyte and embryo development in human and dairy cows (Van Hoeck et al., 2013; Leroy et al., 2015). Higher concentration of NEFA within the oocytes and in the microenvironment of the embryo is detrimental for their subsequent development and competence acquisition (Leroy et al., 2008). Additionally, an increased level of NEFA in the microenvironment of oocytes can affect gene expression, alter the DNA methylation level of imprinted genes and thus can change the fate of the resultant blastocyst (Van Hoeck et al., 2015). Recently, a study explored the effect of NEFA concentration by exposing oocytes and embryos to a high concentration of oleic (OA), palmitic (PA), and stearic (SA) acid and also performed pyrosequencing for targeted DNA methylation analysis. The sequencing results revealed that most of the genes (*SIRT1, MAD2L1*, *FAM3C*, *CDC7*, *CD47*, *HERC5*, *HSPD1*, *SCP2*, *UBL3*, *ID3*, *GPCPD1*, *CYP11A1*, *PDCD10*, *SCP2*, *KRT19*, *GBP4*, *CCL17*, *SC4MOL*, and *TP53*) related to apoptosis, embryo implantation, gene transcription, immune response and metabolism have an altered expression (Desmet et al., 2016). Thus, a balanced level of fatty acid is necessary to ensure the quality of developing oocytes in mammals which can be useful for the successful maturation of oocytes for monospermic fertilization.

## The Signaling Roles of Lipid Molecules During Oocyte Maturation

All the biological membranes are constituted by different lipid molecules and their derivatives, however, the distribution of lipid contents varies in each developmental stage of mammalian oocytes. The analysis of the porcine ovary revealed the differences in compositions of glycerophospholipids, sphingolipids, and cholesterol derivatives between somatic cells and FFs (Uzbekova et al., 2015). The localization and intensity of some lipid derived molecules like phosphatidylinositol and arachidonic acid continuously vary between pre- and post-ovulated stages in mouse ovaries indicating their diverse role in oogenesis (Campbell et al., 2012). The catabolite products of glycerophospholipids and sphingolipids such as diacylglycerols (DGs) (Eichmann and Lass, 2015), unsaturated FAs (Choi et al., 2019), lysophosphatidic acids (LPA) (Jesionowska et al., 2015), and ceramides (Buschiazzo et al., 2011) perform signaling functions, stimulate cell proliferation, migration, differentiation and survival.

The role of these lipid derivatives in oocyte maturation and developmental competence has been recently studied in various mammalian species. LPA receptors perform an essential role in promoting meiotic maturation and also improve the pre-implantation embryo developmental competence in mouse, bovine and porcine oocytes (Komatsu et al., 2006; Jo et al., 2014; Zhang et al., 2015). Addition of LPA in cultured bovine and porcine oocytes stimulated the expression of anti-apoptotic B-cell lymphoma 2 (BCL2) mRNA and decreased BAX-BCL2 expression (Boruszewska et al., 2015). LPA is also a major regulator of oocyte maturation by modulating the other molecules in a cascade manner. It regulates the concentration of cAMP which induces the germinal vesicle breakdown (GVBD) by activating MAPK and adenylyl cyclase (AC) pathway (Villa-Diaz and Miyano, 2004; Yamashita et al., 2009). It also modulates the activity of PCNA (Proliferating cell nuclear antigen) which affects the development of ovarian follicles. More importantly, LPA regulates the activity of some important enzymes including ACSL3 (Long-chain-fatty acid-CoA ligase 3) and ACADL (Acyl-CoA dehydrogenase, long-chain) that are involved in fatty acid oxidation and improved oocyte metabolism (Dunning et al., 2014a). LPA is also involved in stimulating the uPA (urokinase plasminogen activator) and uPAR (urokinase plasminogen activator receptor) in porcine oocytes. Addition of LPA (30 uM) resulted in higher uPA and uPAR in CCs but not in oocytes, suggesting that LPA activated MAPK pathway in CCs. Additional investigation of nuclear maturation of the experimental group revealed that LPA and EGF (epidermal growth factor) together have a synergistic effect on the metaphase II stage oocyte nuclear maturation (Hwang et al., 2018).

The orchestrating events of oocyte maturation is executed through multiple signaling pathways and one of them is cyclooxygenase-(COX-2) derived prostaglandin E2 (PGE2), a well-known lipid mediator that is critical for oocyte development. PGE2 directs oocyte maturation in cultured mouse ovaries and inhibition of PGE2 by indomethacin attenuates gonadotropin-induced cumulus cell expansion which further delays the GVBD of oocytes (Neal et al., 1975; Downs and Longo, 1983). *Cox^–/–^* mouse displayed a compromised oocyte maturation and cumulus cell expansion along with the delay of GVBD. While the cultured oocytes of *Cox^–/–^* mouse restores the maturation and expansion of CCs when treated with PEG2 indicating that PEG2 is mandatory for oocyte maturation. PGE2 works in coordination with gonadotropin signaling through its cell surface G-protein-coupled EP2 and EP4 receptors. It stimulates cumulus cell expansion and oocyte meiotic maturation in mouse by impinging cAMP-dependent protein kinase, NF-KB, MAPK, and phosphatidylinositol 3-kinase/Akt pathways (Takahashi et al., 2006).

**FIGURE 2:**
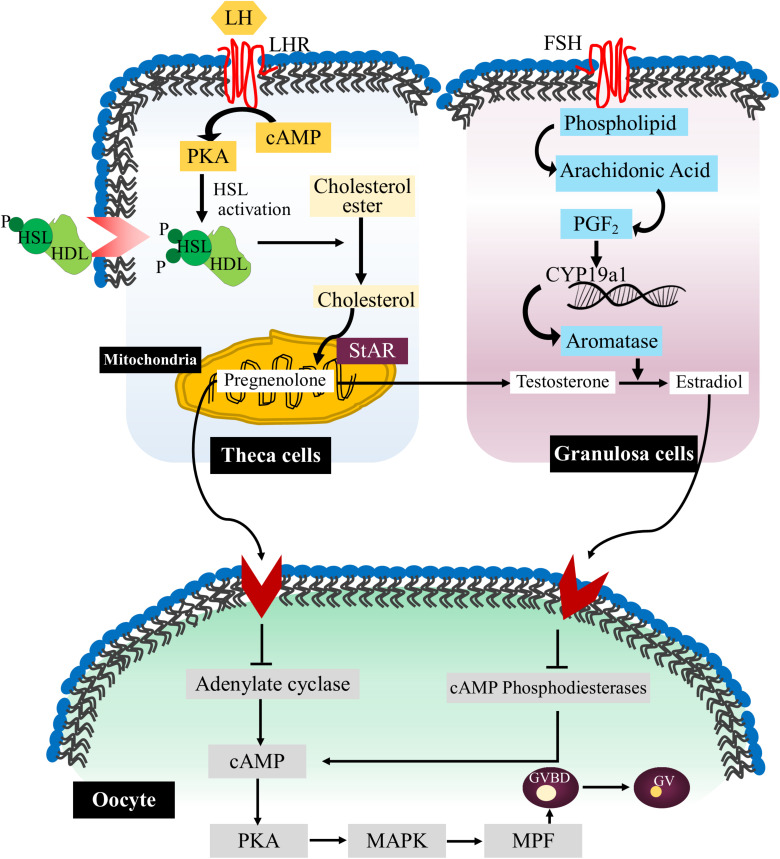
Lipid signaling pathways during oocyte maturation. The flow chart representative diagram explaining the various signaling events occurs during mammalian oocyte maturation. Briefly, luteinizing hormone (LH) through its receptor luteinizing hormone receptor (LHR) stimulates the entry of arachidonic acid in theca cells (TCs) in the form of high density lipoprotein (HDL). Then HSL is activated through cAMP/PKA pathways, and initiates the release of cholesterol from lipid droplets. In the mitochondria, cholesterol molecules are converted into pregnenolone by action of steroidogenic acute regulatory protein (StAR). Furthermore, COX pathway is triggered inside the granulosa cells (GCs) via FSH stimulation which initiates the expression of aromatase and converts testosterone (T) into estradiol (E2). Estradiol further stimulates various pathways in the oocytes which safeguard the degradation of cAMP and concurrent inhibition of maturation-promoting factor (MPF). On the other hand, activation of progesterone (P4) signaling causes inhibition of cAMP production by blocking adenylate cyclase (AC) activity which results in MPF activation and GVBD.

Arachidonic acid is another important polyunsaturated fatty acid (PUFA) that performs various functions in cell behavior and response. It is the part of membrane phospholipid and after releasing from the membrane it is further catabolized into eicosanoids which influences the quality of oocytes (Lapa et al., 2011). Arachidonic acid regulates the dynamics of gap junction by modulating the transfer of small metabolites and regulatory molecules between granulosa cells and oocytes. High level of arachidonic acid can reduce the number of membrane channels while inhibition of arachidonic acid is associated with the increased number of membrane channels (Marandykina et al., 2013). Arachidonic acid also stimulates the activation of protein kinase C (PKC) and MAPK signaling pathways which further promote the production of matrix glycoprotein laminin and Connexin-43 protein in cultured granulosa cells (Jin et al., 2009). Thus, it seems that arachidonic acid pathway in association with kinase activity is necessary for the direct cell-to-cell communication and synergistically modulate paracrine signaling during oocyte maturation (Figure 2).

Gangliosides are also key lipid derivative molecules that belong to the glycosphingolipids family and contain negatively charged sialic acid residues in their carbohydrate moiety (Mirkin et al., 2002). Generally, gangliosides regulate the activity of platelet-derived growth factor receptor (PDGFR), epidermal growth factor receptor (EGFR), and fibroblast growth factor receptor (FGFR) (Xu et al., 2005; Kim et al., 2008). Ganglioside GD1a activates EGFR signaling pathways in vertebrate oocytes and further initiates the resumption of meiosis and expansion of cumulus cells (Uhm et al., 2010). GD1a expression is restricted to COCs and adding GD1a (10 uM) significantly increased the proportion of metaphase II stage of porcine oocytes. Subsequently, exogenous treatment of GD1a initiated the meiotic oocyte maturation and a higher proportion of metaphase I stage oocytes were observed. The addition of GD1a and EGFR can improve the quality and developmental competence of blastocysts in the pre-implantation embryo stage (Kim et al., 2016). Furthermore, sphingolipid also contributes the activation of mTOR pathway and *Tfap2c* translation during mammalian embryogenesis (Chi et al., 2020).

## Regulators of Lipid Metabolism During Oocyte Maturation

It is well known that lipogenesis and lipolysis are important processes during oocyte maturation and embryo development, thus, the regulators of lipid metabolism had been thoroughly studied. Melatonin (N-acetyl-5-methoxytryptamine) which is synthesized during night time from the pineal gland of mammals, has antioxidant properties and regulates various physiological processes such as lipid profile and metabolic syndrome (Kozirog et al., 2011; Stehle et al., 2011; Kitagawa et al., 2012; Calvo et al., 2013; Reiter et al., 2016). Beneficial effects of melatonin on oocyte development have been documented in various mammalian species including sheep, cows, mice, cattle and pigs (Wang et al., 2013, 2014). Recently, the effects of different melatonin concentrations (10*^–^*^3^, 10*^–^*^5^, 10*^–^*^7^, and 10*^–^*^9^ M) on lipid metabolism of the porcine oocytes during *in vitro* maturation have been investigated and a significant increase in the rate of blastocyst formation has been observed with 10*^–^*^9^ M concentration of melatonin compared to other experimental groups. Moreover, the upregulated expression of lipid metabolism-associated genes such as *ACACA*, *FASN*, *PPAR*γ, and *SREBF1* were noted in melatonin treated groups. Subsequently, the role of melatonin in lipolysis has also been evaluated and a greater uptake of FA has been observed in treated groups. Expression of fatty acid oxidation-related genes (CPT1a and b and CPT2 II) was noted to be higher in the melatonin group (Jin et al., 2017). These observations demonstrated the importance of melatonin in lipid metabolism for the acquisition of oocyte developmental competence.

Supplementation of antioxidants during *in vitro* maturation of bovine and other mammalian oocytes is necessary to decrease the generation of reactive oxygen species (ROS) as well as to neutralize the adverse effects on oocyte and embryo development (Jeong et al., 2006; Cicek et al., 2012). Ascorbic acid (AC) and α-tocopherol are well-known antioxidants that are generally used for ROS scavenging both *in vivo* and *in vitro* (Hossein et al., 2007). The addition of these molecules to culture media protects the oocytes and embryos from oxidative damage as well as improves the blastocyst formation. However, AC is sensitive to high temperature and humidity, and thus it should be encapsulated in methyl−β−cyclodextrin (CD) to form an inclusion complex that helps to increase the bioavailability of AC for the developing embryos (Hu et al., 2012). The effects of AC-cyclodextrin complex on the *in vitro* maturation efficiency and lipid metabolism of bovine oocytes has been extensively investigated. Interestingly, no obvious differences had been found in the nuclear maturation of the control and AC-cyclodextrin treated groups, however, AC-cyclodextrin treated oocytes and cumulus cells displayed differential expression of apoptosis and lipid metabolism associated genes. The expression of apoptosis related genes (*BAX* and *BMP15*) were downregulated in AC-cyclodextrin group while lipid metabolism associated gene (*CYP51A1)* expression was upregulated. Though neither blastocyst formation rate nor cleavage rate displayed any significant difference, the increased expression of *CYP51A1* in CCs of AC-cyclodextrin group indicated that AC regulates the cholesterol synthesis during *in vitro* maturation of oocytes (Torres et al., 2019). Overall, these observations might lay the foundation for future improvement of *in vitro* oocyte culture by modifying the metabolism of lipids.

## Abnormal Lipid Metabolism and Oocytes or Zygote Development

### Aging and Oocyte Quality

Aging is known to mediate certain types of complications and pathologies in cellular functions (Kirkwood, 2002). Generally, aging is associated with increased production of ROS and other toxic by-products during aerobic respiration which are likely to affect cellular and mitochondrial genome leading to imbalanced redox activity and aneuploidy (Fragouli et al., 2015). Most of the population consulting reproductive and fertility centers are encountered with women of higher reproductive age. The quality of oocytes from these women is usually compromised and the resulting embryos from these oocytes display poor development (Meldrum et al., 2016; Greaney et al., 2018). Aging can cause reduced oocyte quality, defects in mitochondrial function as well as increased level of mutation and deletion in oocyte mitochondrial DNA (mtDNA) (Wang et al., 2017; Liu et al., 2018; Pasquariello et al., 2019). Oocyte maturation, fertilization potency, cleavage, embryo preimplantation and embryogenesis require high amount of ATP which is generally provided by mtDNA mediated β-oxidation of lipid molecules while increased maternal age is associated with declined energy production efficiency in oocytes and early embryos (Spikings et al., 2007; Eichenlaub-Ritter et al., 2011; Ge et al., 2012). Hence, a prominent change that occurs with age is mitochondrial dysfunction related to the β-oxidation of fatty acids in the oocytes. Therefore, both quality and quantity of mitochondria in the oocytes are important and could be essential indicators for successful fertilization and embryo growth in aged women.

### Effect of Obesity in Oocyte and Zygote Development

Epidemiological findings have suggested that maternal body weight is linked with an elevated risk of cardiovascular and metabolic disorders in the offspring (Lawlor et al., 2012; Reynolds et al., 2013). It is also generally recognized that maternal diet in the periconceptional span can affect oocyte production, embryo development, and offspring health (Gluckman and Hanson, 2004; Connor et al., 2012; Machtinger et al., 2012).

**FIGURE 3:**
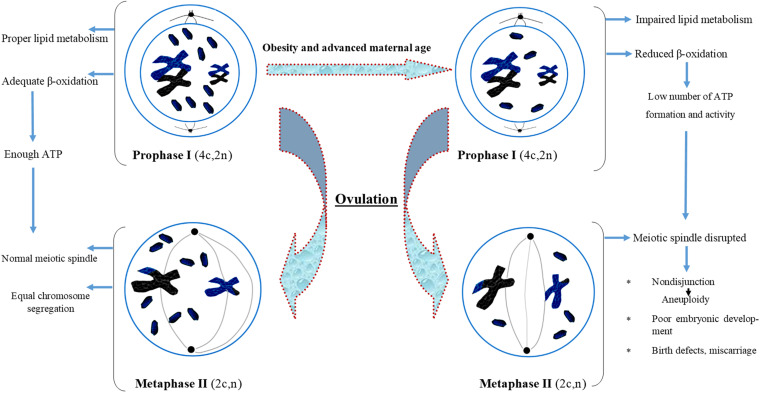
Flow chart diagram describing the effect of obesity and advanced maternal age on oocyte maturation. Obesity and advanced maternal age cause improper lipid metabolism in oocyte which further affect spindle formation, lead to aneuploidy and poor embryonic development.

The ovarian follicular environment is changed in obese women with elevated levels of glucose, triglycerides, and insulin which can have phenotypic effects on the oocytes (Robker et al., 2009; Valckx et al., 2012). Specifically, overweight and obese women face problem of producing fewer oocytes than normal body mass index (BMI) women. The oocytes from overweight and obese women are less likely to enter the blastocyst phase. Studies have also documented that embryos from overweight and obese women display impaired developmental and metabolic phenotypes. The major metabolic anomalies exhibited by these embryos include elevated endogenous triglyceride content, reduced glucose intake and altered amino acid metabolism profile. These finding provide clear evidences of a correlation of maternal diet, periconceptional environment, and oocyte development, which may have long-term health consequences in the offspring (Leary et al., 2015). Overall, these findings pointed toward the possibility of some altered lipid metabolism and age-dependent oocyte development which may be impaired with the growing age. We have also presented all the possible effects of aging and impaired metabolism in the form of a flow chart diagram in Figure 3.

Maternal diet-induced obesity can alter mitochondrial distribution, hyperpolarization of mitochondrial membrane, oxidized redox, and oxidative stress in both oocytes and zygotes. It is reported that 46% of obese mothers experienced the failure of embryo development at the blastocyst stage due to mitochondrial oxidative stress (Zhang et al., 2020). The lack of blastocysts in obese females is not attributed to anovulation but more likely is due to the increased embryonic death. Furthermore, it was reported that an obesogenic diet raised the concentration of serum fatty acids and oviductal leptin. Similarly, high exposure of oocytes and embryos to the obese reproductive environment was correlated with qualitative and quantitative changes in mitochondria, oxidized redox status, increased oxidative load, and impaired antioxidant ability (Igosheva et al., 2010). Thus impaired mitochondrial metabolism of oocytes or early embryos resulting from prolonged exposure to nutrients before and during pregnancy could be responsible for the adverse reproductive outcomes in obese women. Further analysis of mitochondrial functions in oocytes and embryos is required, particularly, of obesity-related alterations in mitochondrial gene expression that control energy metabolism.

### Polycystic Ovary Syndrome and Lipid Metabolism

Polycystic ovary syndrome (PCOS) is one of the complex and most prevalent endocrine disorder in women at reproductive age (Neven et al., 2018). PCOS is manifested with dilute or chronic anovulation, polycystic ovaries, and hyperandrogenism (Balen et al., 2016). Functional inactivation and mutations in the genes that affect steroid hormone functions such as *AR* (androgen receptor) and *SHBG* (sex hormone-binding globulin) have been reported with PCOS in various ethnic groups (Gottlieb et al., 2004; Wickham et al., 2011). Similarly, the distorted function of genes that are necessary for the synthesis of gonadotropin hormone is an important cause of PCOS. The variations in the two most well-known genes, *FSHR* (follicle-stimulating hormone receptor), and *AMH* (anti-mullerian hormone), are associated with PCOS (Wu et al., 2014; Gorsic et al., 2017). On the other hand, genes that are essential for ovarian and adrenal steroidogenesis also play important role in the etiology of PCOS. The defective function of these genes is manifested through the endocrine system which aggravates an elevated level of androgen resulting in PCOS. Thus, the variants in ovarian and adrenal steroidogenesis synthesis genes such as *CYPA1A*, *CYP11A1*, *CYP11B2*, *CYP17A1*, *CYP1A1*, *CYP21A2*, *CYP3A7*, and *CYP19A1* are involved in PCOS (Zhao et al., 2003; Esinler et al., 2008; Unsal et al., 2009; Reddy et al., 2014; Mykhalchenko et al., 2017). Furthermore, mutations and polymorphisms in some other genes including *FTO*, *CAPN10*, *INS*, *INSR*, *SRD5A2*, and *SRD5A1* are also assumed to be associated with PCOS in certain ethnic groups (Goodarzi et al., 2006; Baillargeon and Carpentier, 2007; Cai et al., 2014). Most of these genes are involved in lipid metabolism and androgen synthesis, suggesting that impaired lipogenesis and lipolysis can be an important factor in understanding the etiology of PCOS. We have summarized the reported genes found to be associated with PCOS in Table 2.

**TABLE 2:** Most common genes associated with PCOS.

Gene	Chr. location	Type of mutations	Function	PCOS association	References
*AR*	X	X inactivation	Involved in AR signaling pathway	Associated	Gottlieb et al., 2004
*SHBG*	17	Polymorphism rs727428(C:T)	Control the level of sex hormones	Associated	Wickham et al., 2011
*FSHR*	2	Polymorphism rs6165(997 A/G)	Endocrine reproductive system	Associated	Esinler et al., 2008
					and Unsal et al., 2009
*AMH*	19	Polymorphism rs149082963(254T/G)	Marker of ovarian reserve	Associated	Gorsic et al., 2017
*CYPA1A*	15	Polymorphism rs4646903(6235 T/C)	Metabolization of estrogen	Associated	Esinler et al., 2008
					and Unsal et al., 2009
*CYP11A1*	15	repeat polymorphism (tttta)n	Steroid synthesis	Associated	Reddy et al., 2014
*CYP11B2*	8	Polymorphism rs1799998(–344C/T)	Aldosterone synthetases	Associated	Zhao et al., 2003
*CYP17A1*	10	Polymorphism in promoter rs743572(-34 T/C)	Steroidogenesis enzyme	Associated	Unsal et al., 2009
*CYP1A1*	15	Polymorphism rs1048943(A > G)	Transport pathways of estrogens	Associated	Unsal et al., 2009
*CYP21A2*	6	Polymorphism (V281L and P30L)	Steroid 21-hydroxylase,	Associated	Zhao et al., 2003
*CYP3A7*	7	CYP3A7*1C (promoter region)	Metabolism of DHEAS	Associated	Unsal et al., 2009
*CYP19A1*	15	Polymorphism rs2414096(A/G)	Biosynthesis of cholesterol	Associated	Mykhalchenko et al., 2017
*FTO*	16	Polymorphism rs9939609(A/T)	Lipid Metabolism	Associated	Cai et al., 2014
*CAPN10*	2	Polymorphism rs2975760(4841 T/C)	Insulin action	Associated	Unsal et al., 2009
*INS*	11	VNTR I/I, I/III, and III/III linkage analysis	Production of androgen	Controversial	Baillargeon and Carpentier, 2007
*INSR*	19	Polymorphism rs1799817(3364 T/C)	Insulin receptor	Debatable	Unsal et al., 2009
*SRD5A2*	2	Polymorphism rs523349(c.265C/T)	Androgen biosynthesis	Associated	Goodarzi et al., 2006
*SRD5A1*	5	Polymorphism rs3822430(c.309A/G)	Reduction of testosterone into androgen	Debatable	Goodarzi et al., 2006

**FIGURE 4:**
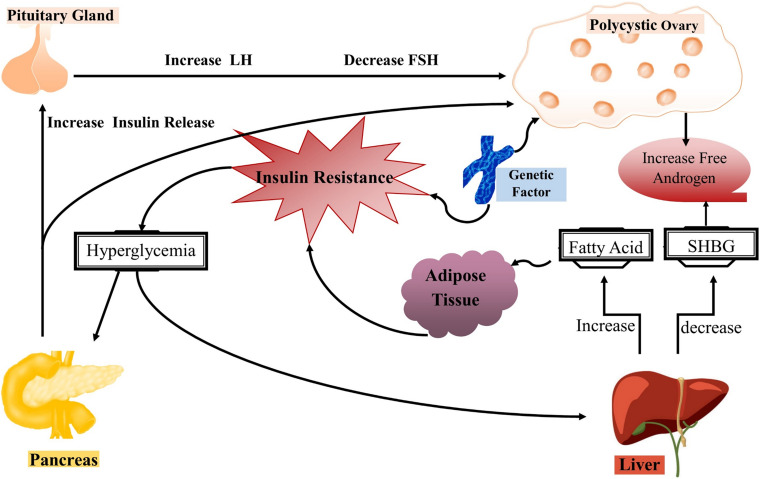
Pathophysiology of polycystic ovary syndrome (PCOS). The representative illustration of complex interactions underlying the pathophysiology of PCOS. Generally, insulin resistance occurs in PCOS that further causes hyperinsulinemia which is responsible for the majority of changes in PCOS women. Skeletal muscles and adipose tissues become insulin resistance with reduced glucose uptake and higher lipolysis, while the ovaries remain insulin sensitive. However, hyperinsulinemia occurs as a compensatory response to insulin resistance and stimulates enhanced production of androgens from ovaries and adrenal glands in PCOS women. In short, excess insulin stimulates increased androgen production in ovarian theca cells in response to luteinizing hormone, resulting in follicular arrest and anovulation. On the other hand, hyperinsulinemia causes suppression of hepatic sex hormone-binding globulin (SHBG) production and leads to hyperandrogenemia.

In mammalian females, evolutionary mechanisms integrate nutritional, environmental and hormonal cues to ensure the successful reproduction under normal energetic conditions, beware that alterations in these events can affect the oocyte development and quality. Impaired metabolism such as obesity affects the reproductive health of females and also causes compromised fertility that can lead to PCOS in severe cases. The role of obesity in PCOS is well-reviewed in many articles and they have regarded obesity as a cause of infertility in females (Jeanes and Reeves, 2017; Silvestris et al., 2018; Ajmal et al., 2019). The impact of obesity on ovulatory disorders is attributed to a dysregulated endocrine system which in turn reduces the ovulation homeostasis (Pasquali et al., 2003). The impaired endocrine system further causes gonadotropin secretion, enhanced aromatization of androgens to estrogens as well as insulin resistance. Keeping in view all these linked metabolic pathways and their involvement in PCOS, we have summarized the role of each pathway in Figure 4. However, still the complete mechanism underlying PCOS is unknown and it is suggested that lipid metabolism including phospholipids, free fatty acids and epigenetic factors such as methylation alterations can be new factors in understanding the complete etiology of PCOS.

The lipidomics approach is utilized to investigate the concentration of lipid profile in PCOS women. A comprehensive study was carried out to evaluate the profile of serum lipids in both obese and lean women with PCOS. The increased concentration of phosphatidylcholine (PC) and concomitantly low level of lysophospholipid (LPC) were observed in the obese PCOS group. Additionally, decreased concentration of PUFA such as linoleic acid, docosahexaenoic acid (DHA) and a higher level of saturated fatty acids were evident in obese PCOS women. Interestingly, downstream bioactive compounds that are generated by PUFA metabolism were higher in the serum of control women than in the lean and obese PCOS women (Li et al., 2017).

Recently, another study was conducted on PCOS women to explore the role of epigenetic factors in PCOS. The study revealed that around 92 differentially expressed genes were unique in PCOS woman granulosa cells and bioinformatic analysis demonstrated that the synthesis of lipids and steroids was stimulated in these cells. Furthermore, the 5-methylcytosine analysis indicated a significant reduction of global methylation in PCOS granulosa cells. Interestingly, hypomethylation of promoter regions was evident in the genes that are related to lipid and steroid metabolism (Pan et al., 2018). Overall, these findings suggested that aberrant DNA methylation of lipid and steroid synthesis genes may lead to dysregulation of steroid metabolism which can stimulate the excess production of androgens and cause PCOS in women.

Emerging evidences also portrayed that gut metabolic abnormalities are also involved in the pathogenesis of PCOS (Zhao et al., 2020). Gut microbes (GM) have the ability to alter the lipid metabolism associated gene expression of host organism causing adiposity and weight gain as well as impacting metabolic, inflammatory pathways and the gut-brain axis (Ussar et al., 2015; Bauer et al., 2016). The microenvironment of the human GM consists of about 1000-1500 species of bacteria (Gill et al., 2006). The dominant species include Prevotella, Porphyromonas, Clostridium, and Eubacterium while the less abundant ones are actinomycetes, proteobacteria, and methanogenic archaea (Lozupone et al., 2012). Dysbiogenesis of GM is associated with PCOS and women with PCOS contain fewer species of GM as compared to normal ones (Torres et al., 2018). Study of GM on the host’s PCOS phenotype revealed that mice transplanted with fecal samples from PCOS patients developed insulin resistance, had an increased number of ovarian cyst-like follicles, a decreased corpus luteum, higher levels of testosterone, luteinizing hormone and produced fewer pups than control mice (Qi et al., 2019). Lindheim et al. (2017) reported that PCOS patients also harbor lower levels of Tenericutes ML615J-28 and Bacteroidetes S24-7 than the normal women. Mice with letrozole-induced PCOS showed decreased genus of GM while number of Firmicutes (closely related to obesity and metabolisches Syndrom) increased (Kelley et al., 2016). Dysbiogenesis of GM also affects the level of ASH (appetite-stimulating hormone) that regulates the secretion of GnRH and sex hormones. The metastatic analysis of PCOS patients displayed lower level of ASH which in turn activates higher expression of aromatase CYP19A1 (Gao et al., 2016). Reduced level of ASH is also a major cause of abnormal metabolism of short chain fatty acids (SCFAs). Thus, it can be inferred that dysbiogeneis of GM may be involved in the occurrence and development of PCOS and the routine investigation of GM may present a new insight to treat PCOS in future.

Micro-RNAs (miRNAs) are small non-coding RNA molecules, approximately consisting of 20–23 nucleotides, have the ability to inhibit the translation of their target genes by a well-known RNA interference (RNAi) mechanism (Trujillo et al., 2010). These small RNA molecules are responsible for a broad range of physiological processes such as proliferation (Sirotkin et al., 2010), differentiation (Qu et al., 2014), and cell metabolism (Sang et al., 2013). Aberrant expression of miRNAs has been linked to the occurrence and development of PCOS (Cai et al., 2017). For example, normal expression of miR-145 is required to inhibit the activation of MAPK/ERK signaling pathways through binding with insulin receptor substrate 1 (IRS1). However, the granulosa cells of PCOS women displayed a lower expression of miR-145 that in turn activates IRS1/MAPK/ERK pathways causing dysregulation of granulosa cell proliferation in PCOS women (Cai et al., 2017). MiR-126-5p and miR-29a-5p expression were also found to be lower in the granulosa cells of PCOS women which induce apoptosis of granulosa cells through the Klotho-associated signaling pathway (Mao et al., 2018). Studies reported that the dysregulated function of miRNAs such as miR-320a (Zhang et al., 2017) and miR-509-3p (Huang et al., 2016) in granulosa cells is associated with aberrant metabolism of estrogen and estradiol secretion which are the common characteristics of PCOS. Downregulated expression of miR-92a and miR-92b was observed in theca cells of PCOS women and gene target analysis confirmed that both of these miRNAs regulate the expression of GATA6, whose protein products regulate the activity of human CYP17 promoter which is involved in lipid metabolism (Lin et al., 2015).

A relationship between miRNAs level in ovarian FF, development and maturation of oocytes has been suggested (Matsuno et al., 2019). Microarray profiling of human FF revealed a significantly increased expression of hsa-miR-9,-18b,-32,-34c, and miR-135a in PCOS group and normal expression of these miRNAs is required for a balanced carbohydrate and lipids metabolism (Roth et al., 2014). Another study reported that around 100 miRNAs showed differential expression in FF in PCOS females. Of note, miR-132 and miR-320 which are the important regulators of steroid synthesis were significantly downregulated implying their involvement in the pathogenesis of PCOS (Sang et al., 2013). Further study reported that let-7b and miR-140 were downregulated while miR-30a was upregulated in the FF of PCOS women (Scalici et al., 2016). These short RNAs are involved in the regulation of androgen production and their dysregulated expression in FF caused excessive androgen production which in turn lead to poor follicular development and ovulatory failure in PCOS (Sang et al., 2013).

Ovarian FF of mammals also contains exosome bound miRNAs. These exosomes encapsulated miRNAs are involved in regulating follicular development, ovulation, and early embryonic development (da Silveira et al., 2012, 2015; Gross et al., 2012; Sang et al., 2013; Di Pietro, 2016; Machtinger et al., 2016; Martinez et al., 2018). Several reports suggested that exosome encapsulated miRNAs also maintain intracellular communication between ovarian function and follicle development (Sun et al., 2019). Furthermore, bioinformatics based analysis identified 167 upregulated and 245 downregulated circRNAs from exosomes of FF obtained from PCOS patients (Wang et al., 2019). Thus, due to ill defined etiology of PCOS, understanding the role of miRNAs in the pathogenesis of PCOS may provide novel therapeutic strategies for the treatment of PCOS.

## Current Challenges and Future Directions

According to recent world report by the International Committee for Monitoring ART (assisted reproductive technology), the use of ATR has increased dramatically worldwide over the past two decades (Dyer et al., 2016), however, its efficiency is still low in terms of live birth. For instance, 4.45 cycles are required for one live birth event following *in vitro* fertilization (IVF) across all age groups. It is estimated that less than 7% of retrieved oocytes develop into normal embryos that yields a live birth. The low success rate manifests the poor knowledge of the molecular determinants of oocyte and embryo viability (Montani et al., 2019). To further improve the IVF efficiency and to get more information about the factors affecting the oocyte quality, recent studies are focusing more on FF composition. Studies have focused on hormones, growth factors and ROS profile of the FF and have linked them with oocyte quality (Reinthaller et al., 1987; Basuray et al., 1988; Enien et al., 1995; Mantzoros et al., 2000; De Placido et al., 2006). Recently, the role of lipid metabolism in oocyte maturation and quality control is also evident. It has been demonstrated that carnitine palmitoyltransferase I (*CPT1B)* plays an important role in the β-oxidation of fatty acids during oocyte maturation and embryo development (Ye et al., 2010). *Cpt1b* expression was noted in murine COCs and its high expression was observed after injection of hormones that stimulate oocyte maturation and ovulation. It was also found that the level of β-oxidation was increased during oocyte maturation, as measured by the production of ^3^H_2_O in the medium. Furthermore, inhibition of β-oxidation with etomoxir during oocyte maturation caused retarded embryo development in 3–5 day of post-coitum and thus it was deduced that β-oxidation is essential for oocyte developmental competence. Similarly, supplementation of L-Carnitine during oocyte maturation increased β-oxidation, improved developmental competence and also assisted in the 2-cell cleavage in the absence of carbohydrates supply (Dunning et al., 2010). Thus, understanding of lipid metabolism of oocyte and embryo development in the surrounding microenvironment will pave the way for non-invasive ART in the future.

Lipid profiling of biological systems has been an intensive area of research since 1960s (Montani et al., 2019). However, the research has become more prominent with the emergence of lipidomics. It is a comprehensive understanding of the influence of all lipids on the biological system with respect to cell signaling, transcriptional and translational modulation, and response to environmental changes. The success of lipidomics is attributed to the powerful detection and quantification method that involves interdisciplinary integration of biological, analytical, statistical and bioinformatics approaches (Triebl et al., 2017). On the other hand, non-invasive tools are much more desirable to study the change in lipid profiles with the developmental stages. Thus, recently microprobe capillary electrophoresis (Onjiko et al., 2016), single-cell capillary electrophoresis high-resolution mass spectrometry (CE-HRMS) and multiple reaction monitoring (MRM) are reported for lipid profiling (Onjiko et al., 2017). For example, MRM-profiling is suitable for the analysis of oocytes and embryos since it avoids sample chromatographic dilution, and separation of untargeted lipidomics and has been successfully employed for lipid profiling of 2–6 cell blastocyst (de Lima et al., 2018). Additionally, Nomarski interference differential contrast (NIC) approach was also successfully utilized to detect lipid alterations in porcine oocytes as an appropriate and non-invasive technique to evaluate the lipid content of a single oocyte before or after *in vitro* maturation (Prates et al., 2013). Similarly, coherent anti-Stokes Raman scattering (CARS) microscopy for the comparative quantification of lipid content in different mammalian oocytes at different developmental stages with only ∼2 min of laser exposure without detrimental effects was employed as a new non-invasive tool (Jasensky et al., 2016). Thus, the non-invasive tools for complete lipid profiles in a single cell will provide new approaches that will greatly improve the understanding of the lipid metabolism during oocyte maturation and embryo development.

It is well known that lipid dysregulation has a strong correlation with both male and female infertility (Pocate-Cheriet et al., 2020). Complete information regarding the biochemical, metabolic and molecular pathways of lipids in oocyte maturation and early embryonic development is scarce (Dubeibe Marin et al., 2019). To be noted, the foundation for existing ART is based on animal studies. A wide range of animal models including vertebrates (Xenopus, Zebrafish, mouse, and bovine), urochordates (ascidian), and protostomes are used for egg and sperm studies, which greatly improved our understating of oocyte maturation and embryo development (Khoury et al., 2018). The basic plan of the early development is mostly similar, however, the intervening events differ among species. For instance, the pig and ruminant oocytes depend more on lipid metabolism, whereas, rodent and human oocytes mostly depend on glucose and pyruvate metabolism (Dalbies-Tran et al., 2020). Due to the procedural and ethical barriers in using human oocytes, still there is need to define the best animal model for translational studies of oocyte lipidomics.

Recent advancement in organoid technology is revolutionizing knowledge about the function of biological systems. This organoid technology is recently being used to study clinical applications, toxicology studies and drug discoveries (Xiao et al., 2017; Mancini and Pensabene, 2019). The use of microfluidics technology has already enabled researchers to study organ-on-chip that may lead to create multi-organoid-on-a chip plate form and even human-on-a-chip plate form (Heidari-Khoei et al., 2020). Thus, in future, it may become a powerful tool for the understanding and exploration of lipid metabolism of oocytes and their microenvironment.

## Conclusion

Lipid droplets are active molecules having an important role in the lipid metabolism. Generally, lipid droplets consist of neutral lipids, mostly triglyceride (TG) and cholesterol ester (CE), and offers substrates for energy production, signaling lipids and membrane components. Usually, lipid droplets are stored in the cytoplasm during mammalian oogenesis, although for unknown reasons the exact contents of lipid droplets vary widely among species. Numerous studies have demonstrated the important functions of lipid droplets and free fatty acids in oocyte growth and development. Still, this topic is under extensive investigation and novel functions of these molecules in oocyte development and competence acquisition are being reported on daily basis. Thus, to improve the efficiency of ART in females, focus on the regulation of lipid metabolism during oogenesis is required. In short, extensive information is available about the prevalence of fatty acids, triglycerides and lipoproteins in the microenvironment of COC and researchers should pay more attention to investigate their exact function in oocyte maturation and embryo development.

## Author Contributions

QS and XJ conceived the review. RK and XJ collected the information and wrote the manuscript. UH and QS involved in modification of the manuscript. All authors contributed to the article and approved the submitted version.

## Conflict of Interest

The authors declare that the research was conducted in the absence of any commercial or financial relationships that could be construed as a potential conflict of interest.

## References

[B1] Aardema H. Lolicato F. Van De Lest C. H. Brouwers J. F. Vaandrager A. B. Van Tol H. T. (2013). Bovine cumulus cells protect maturing oocytes from increased fatty acid levels by massive intracellular lipid storage. *Biol. Reprod.* 88:164. 10.1095/biolreprod.112.106062 23616596

[B2] Aardema H. Vos P. L. Lolicato F. Roelen B. A. Knijn H. M. Vaandrager A. B. (2011). Oleic acid prevents detrimental effects of saturated fatty acids on bovine oocyte developmental competence. *Biol. Reprod.* 85 62–69. 10.1095/biolreprod.110.088815 21311036

[B3] Ajmal N. Khan S. Z. Shaikh R. (2019). Polycystic ovary syndrome (PCOS) and genetic predisposition: a review article. *Eur. J. Obstet. Gynecol. Reprod. Biol. X* 3:100060. 10.1016/j.eurox.2019.100060 31403134 PMC6687436

[B4] Apparicio M. Ferreira C. R. Tata A. Santos V. G. Alves A. E. Mostachio G. Q. (2012). Chemical composition of lipids present in cat and dog oocyte by matrix-assisted desorption ionization mass spectrometry (MALDI- MS). *Reprod. Domest. Anim.* 47(Suppl. 6) 113–117. 10.1111/rda.12003 23279478

[B5] Auclair S. Uzbekov R. Elis S. Sanchez L. Kireev I. Lardic L. (2013). Absence of cumulus cells during in vitro maturation affects lipid metabolism in bovine oocytes. *Am. J. Physiol. Endocrinol. Metab.* 304 E599–E613. 23321473 10.1152/ajpendo.00469.2012

[B6] Baillargeon J. P. Carpentier A. (2007). Role of insulin in the hyperandrogenemia of lean women with polycystic ovary syndrome and normal insulin sensitivity. *Fertil. Steril.* 88 886–893. 10.1016/j.fertnstert.2006.12.055 17559844 PMC3846535

[B7] Balen A. H. Morley L. C. Misso M. Franks S. Legro R. S. Wijeyaratne C. N. (2016). The management of anovulatory infertility in women with polycystic ovary syndrome: an analysis of the evidence to support the development of global WHO guidance. *Hum. Reprod. Update* 22 687–708. 10.1093/humupd/dmw025 27511809

[B8] Basuray R. Rawlins R. G. Radwanska E. Henig I. Sachdeva S. Tummon I. (1988). High progesterone/estradiol ratio in follicular fluid at oocyte aspiration for in vitro fertilization as a predictor of possible pregnancy. *Fertil. Steril.* 49 1007–1011. 10.1016/s0015-0282(16)59952-x 3131157

[B9] Bauer P. V. Hamr S. C. Duca F. A. (2016). Regulation of energy balance by a gut-brain axis and involvement of the gut microbiota. *Cell. Mol. Life Sci.* 73 737–755. 10.1007/s00018-015-2083-z 26542800 PMC11108299

[B10] Boruszewska D. Sinderewicz E. Kowalczyk-Zieba I. Grycmacher K. Woclawek-Potocka I. (2015). The effect of lysophosphatidic acid during in vitro maturation of bovine cumulus-oocyte complexes: cumulus expansion, glucose metabolism and expression of genes involved in the ovulatory cascade, oocyte and blastocyst competence. *Reprod. Biol. Endocrinol.* 13:44. 10.1186/s12958-015-0044-x PMC4438640 25981539

[B11] Bradley J. Pope I. Masia F. Sanusi R. Langbein W. Swann K. (2016). Quantitative imaging of lipids in live mouse oocytes and early embryos using CARS microscopy. *Development* 143 2238–2247. 10.1242/dev.129908 27151947 PMC4920167

[B12] Bradley J. Pope I. Wang Y. Langbein W. Borri P. Swann K. (2019). Dynamic label-free imaging of lipid droplets and their link to fatty acid and pyruvate oxidation in mouse eggs. *J. Cell Sci.* 132:jcs228999. 10.1242/jcs.228999 31182643

[B13] Buschiazzo J. Alonso T. S. Biscoglio M. Antollini S. S. Bonini I. C. (2011). Nongenomic steroid- and ceramide-induced maturation in amphibian oocytes involves functional caveolae-like microdomains associated with a cytoskeletal environment. *Biol. Reprod.* 85 808–822. 10.1095/biolreprod.110.090365 21653896

[B14] Cai G. Ma X. Chen B. Huang Y. Liu S. Yang H. (2017). MicroRNA-145 negatively regulates cell proliferation through targeting IRS1 in isolated ovarian granulosa cells from patients with polycystic ovary syndrome. *Reprod. Sci.* 24 902–910. 10.1177/1933719116673197 27799458

[B15] Cai X. Liu C. Mou S. (2014). Association between fat mass- and obesity-associated (FTO) gene polymorphism and polycystic ovary syndrome: a meta-analysis. *PLoS One* 9:e86972. 10.1371/journal.pone.0086972 24466303 PMC3899374

[B16] Calvo J. R. Gonzalez-Yanes C. Maldonado M. D. (2013). The role of melatonin in the cells of the innate immunity: a review. *J. Pineal Res.* 55 103–120. 10.1111/jpi.12075 23889107

[B17] Campbell D. I. Ferreira C. R. Eberlin L. S. Cooks R. G. (2012). Improved spatial resolution in the imaging of biological tissue using desorption electrospray ionization. *Anal. Bioanal. Chem.* 404 389–398. 10.1007/s00216-012-6173-6 22706326

[B18] Chi F. Sharpley M. S. Nagaraj R. Roy S. S. Banerjee U. (2020). Glycolysis-Independent glucose metabolism distinguishes TE from ICM fate during mammalian embryogenesis. *Dev. Cell* 53 9–26. 10.1016/j.devcel.2020.02.015 32197068 PMC7289320

[B19] Choi S. Yoo Y. J. Kim H. Lee H. Chung H. Nam M. H. (2019). Clinical and biochemical relevance of monounsaturated fatty acid metabolism targeting strategy for cancer stem cell elimination in colon cancer. *Biochem. Biophys. Res. Commun.* 519 100–105. 10.1016/j.bbrc.2019.08.137 31481234

[B20] Cicek N. Eryilmaz O. G. Sarikaya E. Gulerman C. Genc Y. (2012). Vitamin E effect on controlled ovarian stimulation of unexplained infertile women. *J. Assist. Reprod. Genet.* 29 325–328. 10.1007/s10815-012-9714-1 22302530 PMC3309992

[B21] Connor K. L. Vickers M. H. Beltrand J. Meaney M. J. Sloboda D. M. (2012). Nature, nurture or nutrition? Impact of maternal nutrition on maternal care, offspring development and reproductive function. *J. Physiol.* 590 2167–2180. 10.1113/jphysiol.2011.223305 22411006 PMC3447158

[B22] da Silveira J. C. Veeramachaneni D. N. Winger Q. A. Carnevale E. M. Bouma G. J. (2012). Cell-secreted vesicles in equine ovarian follicular fluid contain miRNAs and proteins: a possible new form of cell communication within the ovarian follicle. *Biol. Reprod.* 86:71. 10.1095/biolreprod.111.093252 22116803

[B23] da Silveira J. C. Winger Q. A. Bouma G. J. Carnevale E. M. (2015). Effects of age on follicular fluid exosomal microRNAs and granulosa cell transforming growth factor-β signalling during follicle development in the mare. *Reprod. Fertil. Dev.* 27 897–905. 10.1071/rd14452 25945781

[B24] Dalbies-Tran R. Cadoret V. Desmarchais A. Elis S. Maillard V. Monget P. (2020). A comparative analysis of oocyte development in mammals. *Cells* 9:1002. 10.3390/cells9041002 32316494 PMC7226043

[B25] de Lima C. B. Ferreira C. R. Milazzotto M. P. Sobreira T. J. P. Vireque A. A. Cooks R. G. (2018). Comprehensive lipid profiling of early stage oocytes and embryos by MRM profiling. *J. Mass Spectrom.* 53 1247–1252. 10.1002/jms.4301 30325087

[B26] De Placido G. Alviggi C. Clarizia R. Mollo A. Alviggi E. Strina I. (2006). Intra-follicular leptin concentration as a predictive factor for in vitro oocyte fertilization in assisted reproductive techniques. *J. Endocrinol. Invest.* 29 719–726. 10.1007/bf03344182 17033261

[B27] Desmet K. L. Van Hoeck V. Gagne D. Fournier E. Thakur A. O’doherty A. M. (2016). Exposure of bovine oocytes and embryos to elevated non-esterified fatty acid concentrations: integration of epigenetic and transcriptomic signatures in resultant blastocysts. *BMC Genomics* 17:1004. 10.1186/s12864-016-3366-y 27931182 PMC5146907

[B28] Di Pietro C. (2016). Exosome-mediated communication in the ovarian follicle. *J. Assist. Reprod. Genet.* 33 303–311. 10.1007/s10815-016-0657-9 26814471 PMC4785163

[B29] Downs S. M. Longo F. J. (1983). An ultrastructural study of preovulatory apical development in mouse ovarian follicles: effects of indomethacin. *Anat. Rec.* 205 159–168. 10.1002/ar.1092050206 6846867

[B30] Dubeibe Marin D. F. Da Costa N. N. Di Paula Bessa Santana P. De Souza E. B. Ohashi O. M. (2019). Importance of lipid metabolism on oocyte maturation and early embryo development: can we apply what we know to buffalo? *Anim. Reprod. Sci.* 211:106220. 10.1016/j.anireprosci.2019.106220 31785645

[B31] Dumollard R. Carroll J. Duchen M. R. Campbell K. Swann K. (2009). Mitochondrial function and redox state in mammalian embryos. *Semin. Cell Dev. Biol.* 20 346–353. 10.1016/j.semcdb.2008.12.013 19530278

[B32] Dunning K. R. Anastasi M. R. Zhang V. J. Russell D. L. Robker R. L. (2014a). Regulation of fatty acid oxidation in mouse cumulus-oocyte complexes during maturation and modulation by PPAR agonists. *PLoS One* 9:e87327. 10.1371/journal.pone.0087327 24505284 PMC3914821

[B33] Dunning K. R. Cashman K. Russell D. L. Thompson J. G. Norman R. J. Robker R. L. (2010). Beta-oxidation is essential for mouse oocyte developmental competence and early embryo development. *Biol. Reprod.* 83 909–918. 10.1095/biolreprod.110.084145 20686180

[B34] Dunning K. R. Russell D. L. Robker R. L. (2014b). Lipids and oocyte developmental competence: the role of fatty acids and beta-oxidation. *Reproduction* 148 R15–R27. 24760880 10.1530/REP-13-0251

[B35] Dyer S. Chambers G. M. De Mouzon J. Nygren K. G. Zegers-Hochschild F. Mansour R. (2016). International committee for monitoring assisted reproductive technologies world report: assisted reproductive technology 2008, 2009 and 2010. *Hum. Reprod.* 31 1588–1609. 10.1093/humrep/dew082 27207175

[B36] Eichenlaub-Ritter U. Wieczorek M. Lüke S. Seidel T. (2011). Age related changes in mitochondrial function and new approaches to study redox regulation in mammalian oocytes in response to age or maturation conditions. *Mitochondrion* 11 783–796. 10.1016/j.mito.2010.08.011 20817047

[B37] Eichmann T. O. Lass A. (2015). DAG tales: the multiple faces of diacylglycerol–stereochemistry, metabolism, and signaling. *Cell. Mol. Life Sci.* 72 3931–3952. 10.1007/s00018-015-1982-3 26153463 PMC4575688

[B38] Enien W. M. El Sahwy S. Harris C. P. Seif M. W. Elstein M. (1995). Human chorionic gonadotrophin and steroid concentrations in follicular fluid: the relationship to oocyte maturity and fertilization rates in stimulated and natural in-vitro fertilization cycles. *Hum. Reprod.* 10 2840–2844. 10.1093/oxfordjournals.humrep.a135804 8747029

[B39] Esinler I. Aktas D. Otegen U. Alikasifoglu M. Yarali H. Tuncbilek E. (2008). CYP1A1 gene polymorphism and polycystic ovary syndrome. *Reprod. Biomed. Online* 16 356–360. 10.1016/s1472-6483(10)60596-2 18339256

[B40] Ferguson E. M. Leese H. J. (2006). A potential role for triglyceride as an energy source during bovine oocyte maturation and early embryo development. *Mol. Reprod. Dev.* 73 1195–1201. 10.1002/mrd.20494 16804881

[B41] Fragouli E. Spath K. Alfarawati S. Kaper F. Craig A. Michel C. E. (2015). Altered levels of mitochondrial DNA are associated with female age, aneuploidy, and provide an independent measure of embryonic implantation potential. *PLoS Genet* 11:e1005241. 10.1371/journal.pgen.1005241 26039092 PMC4454688

[B42] Gao T. Wu L. Chang F. Cao G. (2016). Low circulating ghrelin levels in women with polycystic ovary syndrome: a systematic review and meta-analysis. *Endocr. J.* 63 93–100. 10.1507/endocrj.ej15-0318 26607017 PMC4975374

[B43] Gautier T. Becker S. Drouineaud V. Menetrier F. Sagot P. Nofer J. R. (2010). Human luteinized granulosa cells secrete apoB100-containing lipoproteins. *J. Lipid Res.* 51 2245–2252. 10.1194/jlr.m005181 20407020 PMC2903810

[B44] Ge H. Tollner T. L. Hu Z. Dai M. Li X. Guan H. (2012). The importance of mitochondrial metabolic activity and mitochondrial DNA replication during oocyte maturation in vitro on oocyte quality and subsequent embryo developmental competence. *Mol. Reprod. Dev.* 79 392–401. 10.1002/mrd.22042 22467220

[B45] Gill S. R. Pop M. Deboy R. T. Eckburg P. B. Turnbaugh P. J. Samuel B. S. (2006). Metagenomic analysis of the human distal gut microbiome. *Science* 312 1355–1359. 16741115 10.1126/science.1124234 PMC3027896

[B46] Gluckman P. D. Hanson M. A. (2004). Living with the past: evolution, development, and patterns of disease. *Science* 305 1733–1736. 10.1126/science.1095292 15375258

[B47] Goodarzi M. O. Shah N. A. Antoine H. J. Pall M. Guo X. Azziz R. (2006). Variants in the 5alpha-reductase type 1 and type 2 genes are associated with polycystic ovary syndrome and the severity of hirsutism in affected women. *J. Clin. Endocrinol. Metab.* 91 4085–4091. 10.1210/jc.2006-0227 16849416

[B48] Gorsic L. K. Kosova G. Werstein B. Sisk R. Legro R. S. Hayes M. G. (2017). Pathogenic anti-mullerian hormone variants in polycystic ovary syndrome. *J. Clin. Endocrinol. Metab.* 102 2862–2872. 10.1210/jc.2017-00612 28505284 PMC5546867

[B49] Gottlieb B. Beitel L. K. Wu J. H. Trifiro M. (2004). The androgen receptor gene mutations database (ARDB): 2004 update. *Hum. Mutat.* 23 527–533. 10.1002/humu.20044 15146455

[B50] Greaney J. Wei Z. Homer H. (2018). Regulation of chromosome segregation in oocytes and the cellular basis for female meiotic errors. *Hum. Reprod. Update* 24 135–161. 10.1093/humupd/dmx035 29244163

[B51] Gross J. C. Chaudhary V. Bartscherer K. Boutros M. (2012). Active Wnt proteins are secreted on exosomes. *Nat. Cell Biol.* 14 1036–1045. 10.1038/ncb2574 22983114

[B52] Gunesdogan U. Surani M. A. (2016). Developmental competence for primordial germ cell fate. *Curr. Top. Dev. Biol.* 117 471–496. 10.1016/bs.ctdb.2015.11.007 26969996

[B53] Heidari-Khoei H. Esfandiari F. Hajari M. A. Ghorbaninejad Z. Piryaei A. Baharvand H. (2020). Organoid technology in female reproductive biomedicine. *Reprod. Biol. Endocrinol.* 18 020–00621. 10.1186/s12958-020-00621-z PMC7301968 32552764

[B54] Hossein M. S. Hashem M. A. Jeong Y. W. Lee M. S. Kim S. Kim J. H. (2007). Temporal effects of alpha-tocopherol and L-ascorbic acid on in vitro fertilized porcine embryo development. *Anim. Reprod. Sci.* 100 107–117. 10.1016/j.anireprosci.2006.06.013 16860500

[B55] Hu X. Wei B. Li H. Wu C. Bai Y. Xu X. (2012). Preparation of the beta-cyclodextrin-vitamin C (beta-CD-Vc) inclusion complex under high hydrostatic pressure (HHP). *Carbohydr. Polym.* 90 1193–1196. 10.1016/j.carbpol.2012.06.029 22840058

[B56] Huang X. Liu C. Hao C. Tang Q. Liu R. Lin S. (2016). Identification of altered microRNAs and mRNAs in the cumulus cells of PCOS patients: miRNA-509-3p promotes oestradiol secretion by targeting MAP3K8. *Reproduction* 151 643–655. 10.1530/rep-16-0071 27001999

[B57] Hwang S. U. Kim K. J. Kim E. Yoon J. D. Park K. M. Jin M. (2018). Lysophosphatidic acid increases in vitro maturation efficiency via uPA-uPAR signaling pathway in cumulus cells. *Theriogenology* 113 197–207. 10.1016/j.theriogenology.2018.02.020 29554602

[B58] Igosheva N. Abramov A. Y. Poston L. Eckert J. J. Fleming T. P. Duchen M. R. (2010). Maternal diet-induced obesity alters mitochondrial activity and redox status in mouse oocytes and zygotes. *PLoS One* 5:e10074. 10.1371/journal.pone.0010074 20404917 PMC2852405

[B59] Jasensky J. Boughton A. P. Khmaladze A. Ding J. Zhang C. Swain J. E. (2016). Live-cell quantification and comparison of mammalian oocyte cytosolic lipid content between species, during development, and in relation to body composition using nonlinear vibrational microscopy. *Analyst* 141 4694–4706. 10.1039/c6an00629a 27272931

[B60] Jeanes Y. M. Reeves S. (2017). Metabolic consequences of obesity and insulin resistance in polycystic ovary syndrome: diagnostic and methodological challenges. *Nutr. Res. Rev.* 30 97–105. 10.1017/s0954422416000287 28222828

[B61] Jeong Y. W. Park S. W. Hossein M. S. Kim S. Kim J. H. Lee S. H. (2006). Antiapoptotic and embryotrophic effects of alpha-tocopherol and L-ascorbic acid on porcine embryos derived from in vitro fertilization and somatic cell nuclear transfer. *Theriogenology* 66 2104–2112. 10.1016/j.theriogenology.2006.06.007 16876856

[B62] Jesionowska A. Cecerska-Heryc E. Matoszka N. Dolegowska B. (2015). Lysophosphatidic acid signaling in ovarian cancer. *J. Recept. Signal Transduct. Res.* 35 578–584. 26393967 10.3109/10799893.2015.1026444

[B63] Jin J. X. Lee S. Taweechaipaisankul A. Kim G. A. Lee B. C. (2017). Melatonin regulates lipid metabolism in porcine oocytes. *J. Pineal Res.* 62:e12388. 10.1111/jpi.12388 28095627

[B64] Jin Y. M. Tan T. Q. Zhang C. Q. (2009). Effect of arachidonic acid on production of laminin and connexin of granulosa cells from chicken pre-hierarchical follicles. *Asian Australas. J. Anim. Sci.* 22 350–355. 10.5713/ajas.2009.80381

[B65] Jo J. W. Jee B. C. Suh C. S. Kim S. H. (2014). Addition of lysophosphatidic acid to mouse oocyte maturation media can enhance fertilization and developmental competence. *Hum. Reprod.* 29 234–241. 10.1093/humrep/det427 24293550

[B66] Jordanov J. Boyadjieva-Michailova A. (1974). Ultrastructural aspects of lipoprotein passage through oocyte envelopes and storage in ooplasm during avian vitellopoiesis. *Acta Anat.* 89 616–632. 10.1159/000144320 4139869

[B67] Jungheim E. S. Macones G. A. Odem R. R. Patterson B. W. Lanzendorf S. E. Ratts V. S. (2011). Associations between free fatty acids, cumulus oocyte complex morphology and ovarian function during in vitro fertilization. *Fertil. Steril.* 95 1970–1974. 10.1016/j.fertnstert.2011.01.154 21353671 PMC3080431

[B68] Kelley S. T. Skarra D. V. Rivera A. J. Thackray V. G. (2016). The gut microbiome is altered in a letrozole-induced mouse model of polycystic ovary syndrome. *PLoS One* 11:e0146509. 10.1371/journal.pone.0146509 26731268 PMC4701222

[B69] Khoury S. Canlet C. Lacroix M. Z. Berdeaux O. Jouhet J. Bertrand-Michel J. (2018). Quantification of lipids: model, reality, and compromise. *Biomolecules* 8:174. 10.3390/biom8040174 30558107 PMC6316828

[B70] Kim J. W. Park H. J. Chae S. K. Ahn J. H. Do G. Y. Choo Y. K. (2016). Ganglioside GD1a promotes oocyte maturation, furthers preimplantation development, and increases blastocyst quality in pigs. *J. Reprod. Dev.* 62 249–255. 10.1262/jrd.2015-083 26860251 PMC4919288

[B71] Kim S. M. Jung J. U. Ryu J. S. Jin J. W. Yang H. J. Ko K. (2008). Effects of gangliosides on the differentiation of human mesenchymal stem cells into osteoblasts by modulating epidermal growth factor receptors. *Biochem. Biophys. Res. Commun.* 371 866–871. 10.1016/j.bbrc.2008.04.162 18471991

[B72] Kirkwood T. B. (2002). Evolution of ageing. *Mech. Ageing Dev.* 123 737–745. 10.1016/s0047-6374(01)00419-5 11869731

[B73] Kitagawa A. Ohta Y. Ohashi K. (2012). Melatonin improves metabolic syndrome induced by high fructose intake in rats. *J. Pineal Res.* 52 403–413. 10.1111/j.1600-079x.2011.00955.x 22220562

[B74] Komatsu J. Yamano S. Kuwahara A. Tokumura A. Irahara M. (2006). The signaling pathways linking to lysophosphatidic acid-promoted meiotic maturation in mice. *Life Sci.* 79 506–511. 10.1016/j.lfs.2006.01.028 16492384

[B75] Kozirog M. Poliwczak A. R. Duchnowicz P. Koter-Michalak M. Sikora J. Broncel M. (2011). Melatonin treatment improves blood pressure, lipid profile, and parameters of oxidative stress in patients with metabolic syndrome. *J. Pineal Res.* 50 261–266. 10.1111/j.1600-079x.2010.00835.x 21138476

[B76] Lapa M. Marques C. C. Alves S. P. Vasques M. I. Baptista M. C. Carvalhais I. (2011). Effect of trans-10 cis-12 conjugated linoleic acid on bovine oocyte competence and fatty acid composition. *Reprod. Domest. Anim.* 46 904–910. 10.1111/j.1439-0531.2011.01762.x 21366717

[B77] Lawlor D. A. Relton C. Sattar N. Nelson S. M. (2012). Maternal adiposity–a determinant of perinatal and offspring outcomes? *Nat. Rev. Endocrinol.* 8 679–688. 10.1038/nrendo.2012.176 23007319

[B78] Leary C. Leese H. J. Sturmey R. G. (2015). Human embryos from overweight and obese women display phenotypic and metabolic abnormalities. *Hum. Reprod.* 30 122–132. 10.1093/humrep/deu276 25391239

[B79] Leroy J. L. Valckx S. D. Jordaens L. De Bie J. Desmet K. L. Van Hoeck V. (2015). Nutrition and maternal metabolic health in relation to oocyte and embryo quality: critical views on what we learned from the dairy cow model. *Reprod. Fertil. Dev.* 27 693–703. 10.1071/rd14363 25690396

[B80] Leroy J. L. Van Soom A. Opsomer G. Bols P. E. (2008). The consequences of metabolic changes in high-yielding dairy cows on oocyte and embryo quality. *Animal* 2 1120–1127. 10.1017/s1751731108002383 22443723

[B81] Leroy J. L. Vanholder T. Mateusen B. Christophe A. Opsomer G. De Kruif A. (2005). Non-esterified fatty acids in follicular fluid of dairy cows and their effect on developmental capacity of bovine oocytes in vitro. *Reproduction* 130 485–495. 10.1530/rep.1.00735 16183866

[B82] Li S. Chu Q. Ma J. Sun Y. Tao T. Huang R. (2017). Discovery of novel lipid profiles in PCOS: do insulin and androgen oppositely regulate bioactive lipid production? *J. Clin. Endocrinol. Metab.* 102 810–821. 27886515 10.1210/jc.2016-2692 PMC5477809

[B83] Lin L. Du T. Huang J. Huang L. L. Yang D. Z. (2015). Identification of differentially expressed microRNAs in the ovary of polycystic ovary syndrome with hyperandrogenism and insulin resistance. *Chin. Med. J.* 128 169–174. 10.4103/0366-6999.149189 25591557 PMC4837833

[B84] Lindheim L. Bashir M. Münzker J. Trummer C. Zachhuber V. Leber B. (2017). Alterations in gut microbiome composition and barrier function are associated with reproductive and metabolic defects in women with polycystic ovary syndrome (PCOS): a pilot study. *PLoS One* 12:e0168390. 10.1371/journal.pone.0168390 28045919 PMC5207627

[B85] Liu M. J. Sun A. G. Zhao S. G. Liu H. Ma S. Y. Li M. (2018). Resveratrol improves in vitro maturation of oocytes in aged mice and humans. *Fertil. Steril.* 109 900–907. 10.1016/j.fertnstert.2018.01.020 29778389

[B86] Lozupone C. A. Stombaugh J. I. Gordon J. I. Jansson J. K. Knight R. (2012). Diversity, stability and resilience of the human gut microbiota. *Nature* 489 220–230. 10.1038/nature11550 22972295 PMC3577372

[B87] Machtinger R. Combelles C. M. Missmer S. A. Correia K. F. Fox J. H. Racowsky C. (2012). The association between severe obesity and characteristics of failed fertilized oocytes. *Hum. Reprod.* 27 3198–3207. 10.1093/humrep/des308 22968161

[B88] Machtinger R. Laurent L. C. Baccarelli A. A. (2016). Extracellular vesicles: roles in gamete maturation, fertilization and embryo implantation. *Hum. Reprod. Update* 22 182–193. 26663221 10.1093/humupd/dmv055 PMC4755440

[B89] Mancini V. Pensabene V. (2019). Organs-On-Chip models of the female reproductive system. *Bioengineering* 6:103. 10.3390/bioengineering6040103 31703369 PMC6956296

[B90] Mantzoros C. S. Cramer D. W. Liberman R. F. Barbieri R. L. (2000). Predictive value of serum and follicular fluid leptin concentrations during assisted reproductive cycles in normal women and in women with the polycystic ovarian syndrome. *Hum. Reprod.* 15 539–544. 10.1093/humrep/15.3.539 10686193

[B91] Mao Z. Fan L. Yu Q. Luo S. Wu X. Tang J. (2018). Abnormality of Klotho signaling is involved in polycystic ovary syndrome. *Reprod. Sci.* 25 372–383. 10.1177/1933719117715129 28673204

[B92] Marandykina A. Palacios-Prado N. Rimkute L. Skeberdis V. A. Bukauskas F. F. (2013). Regulation of connexin36 gap junction channels by n-alkanols and arachidonic acid. *J. Physiol.* 591 2087–2101. 10.1113/jphysiol.2013.250910 23420660 PMC3634521

[B93] Martinez R. M. Liang L. Racowsky C. Dioni L. Mansur A. Adir M. (2018). Extracellular microRNAs profile in human follicular fluid and IVF outcomes. *Sci. Rep.* 8:17036. 10.1038/s41598-018-35379-3 PMC6242846 30451969

[B94] Matsuno Y. Kanke T. Maruyama N. Fujii W. Naito K. Sugiura K. (2019). Characterization of mRNA profiles of the exosome-like vesicles in porcine follicular fluid. *PLoS One* 14:e0217760. 10.1371/journal.pone.0217760 31188849 PMC6561635

[B95] Matzuk M. M. Burns K. H. Viveiros M. M. Eppig J. J. (2002). Intercellular communication in the mammalian ovary: oocytes carry the conversation. *Science* 296 2178–2180. 10.1126/science.1071965 12077402

[B96] McEvoy T. G. Coull G. D. Broadbent P. J. Hutchinson J. S. Speake B. K. (2000). Fatty acid composition of lipids in immature cattle, pig and sheep oocytes with intact zona pellucida. *J. Reprod. Fertil.* 118 163–170. 10.1530/reprod/118.1.163 10793638

[B97] Meldrum D. R. Casper R. F. Diez-Juan A. Simon C. Domar A. D. Frydman R. (2016). Aging and the environment affect gamete and embryo potential: can we intervene? *Fertil. Steril.* 105 548–559. 10.1016/j.fertnstert.2016.01.013 26812244

[B98] Mirkin B. L. Clark S. H. Zhang C. (2002). Inhibition of human neuroblastoma cell proliferation and EGF receptor phosphorylation by gangliosides GM1, GM3, GD1A and GT1B. *Cell Prolif.* 35 105–115. 10.1046/j.1365-2184.2002.00228.x 11952645 PMC6496818

[B99] Montani D. A. Braga D. Borges E. Jr. Camargo M. Cordeiro F. B. Pilau E. J. (2019). Understanding mechanisms of oocyte development by follicular fluid lipidomics. *J. Assist. Reprod. Genet.* 36 1003–1011. 10.1007/s10815-019-01428-7 31011990 PMC6541691

[B100] Mykhalchenko K. Lizneva D. Trofimova T. Walker W. Suturina L. Diamond M. P. (2017). Genetics of polycystic ovary syndrome. *Expert. Rev. Mol. Diagn.* 17 723–733. 28602111 10.1080/14737159.2017.1340833

[B101] Neal P. Baker T. G. Mcnatty K. P. Scaramuzzi R. J. (1975). Influence of prostaglandins and human chorionic gonadotrophin on progesterone concentration and oocyte maturation in mouse ovarian follicles maintained in organ culture. *J. Endocrinol.* 65 19–25. 10.1677/joe.0.0650019 1141809

[B102] Neven A. C. H. Laven J. Teede H. J. Boyle J. A. (2018). A summary on polycystic ovary syndrome: diagnostic criteria, prevalence, clinical manifestations, and management according to the latest international guidelines. *Semin. Reprod. Med.* 36 5–12. 30189445 10.1055/s-0038-1668085

[B103] Onjiko R. M. Morris S. E. Moody S. A. Nemes P. (2016). Single-cell mass spectrometry with multi-solvent extraction identifies metabolic differences between left and right blastomeres in the 8-cell frog (*Xenopus*) embryo. *Analyst* 141 3648–3656. 10.1039/c6an00200e 27004603 PMC4899105

[B104] Onjiko R. M. Portero E. P. Moody S. A. Nemes P. (2017). In situ microprobe single-cell capillary electrophoresis mass spectrometry: metabolic reorganization in single differentiating cells in the live vertebrate (*Xenopus laevis*) embryo. *Anal. Chem.* 89 7069–7076. 10.1021/acs.analchem.7b00880 28434226 PMC5706767

[B105] Pan J. X. Tan Y. J. Wang F. F. Hou N. N. Xiang Y. Q. Zhang J. Y. (2018). Aberrant expression and DNA methylation of lipid metabolism genes in PCOS: a new insight into its pathogenesis. *Clin. Epigenetics* 10:6. 10.1186/s13148-018-0442-y PMC5767000 29344314

[B106] Pasquali R. Pelusi C. Genghini S. Cacciari M. Gambineri A. (2003). Obesity and reproductive disorders in women. *Hum. Reprod. Update* 9 359–372. 10.1093/humupd/dmg024 12926529

[B107] Pasquariello R. Ermisch A. F. Silva E. Mccormick S. Logsdon D. Barfield J. P. (2019). Alterations in oocyte mitochondrial number and function are related to spindle defects and occur with maternal aging in mice and humans^†^. *Biol. Reprod.* 100 971–981. 10.1093/biolre/ioy248 30476005

[B108] Pepling M. E. Spradling A. C. (2001). Mouse ovarian germ cell cysts undergo programmed breakdown to form primordial follicles. *Dev. Biol.* 234 339–351. 10.1006/dbio.2001.0269 11397004

[B109] Pocate-Cheriet K. Santulli P. Kateb F. Bourdon M. Maignien C. Batteux F. (2020). The follicular fluid metabolome differs according to the endometriosis phenotype. *Reprod. Biomed. Online* 41 1023–1037. 10.1016/j.rbmo.2020.09.002 33046374

[B110] Prates E. G. Alves S. P. Marques C. C. Baptista M. C. Horta A. E. Bessa R. J. (2013). Fatty acid composition of porcine cumulus oocyte complexes (COC) during maturation: effect of the lipid modulators trans-10, cis-12 conjugated linoleic acid (t10,c12 CLA) and forskolin. *In Vitro Cell. Dev. Biol. Anim.* 49 335–345. 10.1007/s11626-013-9624-2 23645468

[B111] Qi X. Yun C. Sun L. Xia J. Wu Q. Wang Y. (2019). Gut microbiota-bile acid-interleukin-22 axis orchestrates polycystic ovary syndrome. *Nat. Med.* 25 1225–1233. 10.1038/s41591-019-0509-0 31332392 PMC7376369

[B112] Qu B. Xia X. Wu H. H. Tu C. Q. Pan X. M. (2014). PDGF-regulated miRNA-138 inhibits the osteogenic differentiation of mesenchymal stem cells. *Biochem. Biophys. Res. Commun.* 448 241–247. 10.1016/j.bbrc.2014.04.091 24792185

[B113] Ranneva S. V. Okotrub K. A. Amstislavsky S. Y. Surovtsev N. V. (2020). Deuterated stearic acid uptake and accumulation in lipid droplets of cat oocytes. *Arch. Biochem. Biophys.* 692:108532. 10.1016/j.abb.2020.108532 32795451

[B114] Reddy K. R. Deepika M. L. Supriya K. Latha K. P. Rao S. S. Rani V. U. (2014). CYP11A1 microsatellite (tttta)n polymorphism in PCOS women from South India. *J. Assist. Reprod. Genet.* 31 857–863. 10.1007/s10815-014-0236-x 24793009 PMC4096885

[B115] Reinthaller A. Deutinger J. Riss P. Muller-Tyl E. Fischl F. Bieglmayer C. (1987). Relationship between the steroid and prolactin concentration in follicular fluid and the maturation and fertilization of human oocytes. *J. In Vitro Fert. Embryo Transf.* 4 228–231. 10.1007/bf01533761 3625003

[B116] Reiter R. J. Mayo J. C. Tan D. X. Sainz R. M. Alatorre-Jimenez M. Qin L. (2016). Melatonin as an antioxidant: under promises but over delivers. *J. Pineal Res.* 61 253–278. 10.1111/jpi.12360 27500468

[B117] Reynolds R. M. Allan K. M. Raja E. A. Bhattacharya S. Mcneill G. Hannaford P. C. (2013). Maternal obesity during pregnancy and premature mortality from cardiovascular event in adult offspring: follow-up of 1 323 275 person years. *BMJ* 347 f4539. 10.1136/bmj.f4539 23943697 PMC3805484

[B118] Robker R. L. Akison L. K. Bennett B. D. Thrupp P. N. Chura L. R. Russell D. L. (2009). Obese women exhibit differences in ovarian metabolites, hormones, and gene expression compared with moderate-weight women. *J. Clin. Endocrinol. Metab.* 94 1533–1540. 10.1210/jc.2008-2648 19223519

[B119] Roth L. W. Mccallie B. Alvero R. Schoolcraft W. B. Minjarez D. Katz-Jaffe M. G. (2014). Altered microRNA and gene expression in the follicular fluid of women with polycystic ovary syndrome. *J. Assist. Reprod. Genet.* 31 355–362. 10.1007/s10815-013-0161-4 24390626 PMC3947080

[B120] Sang Q. Yao Z. Wang H. Feng R. Zhao X. Xing Q. (2013). Identification of microRNAs in human follicular fluid: characterization of microRNAs that govern steroidogenesis in vitro and are associated with polycystic ovary syndrome in vivo. *J. Clin. Endocrinol. Metab.* 98 3068–3079. 10.1210/jc.2013-1715 23666971

[B121] Scalici E. Traver S. Mullet T. Molinari N. Ferrières A. Brunet C. (2016). Circulating microRNAs in follicular fluid, powerful tools to explore in vitro fertilization process. *Sci. Rep.* 6:24976. 10.1038/srep24976 PMC4840336 27102646

[B122] Shibahara H. Ishiguro A. Inoue Y. Koumei S. Kuwayama T. Iwata H. (2020). Mechanism of palmitic acid-induced deterioration of in vitro development of porcine oocytes and granulosa cells. *Theriogenology* 141 54–61. 10.1016/j.theriogenology.2019.09.006 31518729

[B123] Silvestris E. De Pergola G. Rosania R. Loverro G. (2018). Obesity as disruptor of the female fertility. *Reprod. Biol. Endocrinol.* 16:22. 10.1186/s12958-018-0336-z PMC5845358 29523133

[B124] Simpson E. R. Rochelle D. B. Carr B. R. Macdonald P. C. (1980). Plasma lipoproteins in follicular fluid of human ovaries. *J. Clin. Endocrinol. Metab.* 51 1469–1471. 10.1210/jcem-51-6-1469 7440708

[B125] Sirard M. A. (2011). Follicle environment and quality of in vitro matured oocytes. *J. Assist. Reprod. Genet.* 28 483–488. 10.1007/s10815-011-9554-4 21394521 PMC3158252

[B126] Sirotkin A. V. Lauková M. Ovcharenko D. Brenaut P. Mlyncek M. (2010). Identification of microRNAs controlling human ovarian cell proliferation and apoptosis. *J. Cell. Physiol.* 223 49–56. 20039279 10.1002/jcp.21999

[B127] Spikings E. C. Alderson J. St John J. C. (2007). Regulated mitochondrial DNA replication during oocyte maturation is essential for successful porcine embryonic development. *Biol. Reprod.* 76 327–335. 10.1095/biolreprod.106.054536 17035641

[B128] Stehle J. H. Saade A. Rawashdeh O. Ackermann K. Jilg A. Sebesteny T. (2011). A survey of molecular details in the human pineal gland in the light of phylogeny, structure, function and chronobiological diseases. *J. Pineal Res.* 51 17–43. 10.1111/j.1600-079x.2011.00856.x 21517957

[B129] Sturmey R. G. Leese H. J. (2003). Energy metabolism in pig oocytes and early embryos. *Reproduction* 126 197–204. 10.1530/rep.0.1260197 12887276

[B130] Sun X. Ma X. Yang X. Zhang X. (2019). Exosomes and Female Infertility. *Curr. Drug Metab.* 20 773–780. 10.2174/1389200220666191015155910 31749422

[B131] Takahashi T. Morrow J. D. Wang H. Dey S. K. (2006). Cyclooxygenase-2-derived prostaglandin E(2) directs oocyte maturation by differentially influencing multiple signaling pathways. *J. Biol. Chem.* 281 37117–37129. 10.1074/jbc.m608202200 17023426

[B132] Tanghe S. Van Soom A. Nauwynck H. Coryn M. De Kruif A. (2002). Minireview: functions of the cumulus oophorus during oocyte maturation, ovulation, and fertilization. *Mol. Reprod. Dev.* 61 414–424. 10.1002/mrd.10102 11835587

[B133] Tatsumi T. Takayama K. Ishii S. Yamamoto A. Hara T. Minami N. (2018). Forced lipophagy reveals that lipid droplets are required for early embryonic development in mouse. *Development* 145:161893. 10.1242/dev.161893 29475974

[B134] Torres P. J. Siakowska M. Banaszewska B. Pawelczyk L. Duleba A. J. Kelley S. T. (2018). Gut microbial diversity in women with polycystic ovary syndrome correlates with hyperandrogenism. *J. Clin. Endocrinol. Metab.* 103 1502–1511. 10.1210/jc.2017-02153 29370410 PMC6276580

[B135] Torres V. Hamdi M. Maillo V. Urrego R. Echeverri J. J. Lopez-Herrera A. (2019). Ascorbic acid-cyclodextrin complex alters the expression of genes associated with lipid metabolism in bovine in vitro produced embryos. *Reprod. Domest. Anim.* 54 55–62. 10.1111/rda.13311 30120843

[B136] Triebl A. Hartler J. Trötzmüller M. Köfeler H. C. (2017). Lipidomics: prospects from a technological perspective. *Biochim. Biophys. Acta Mol. Cell Biol. Lipids* 8 740–746. 10.1016/j.bbalip.2017.03.004 28341148 PMC6013030

[B137] Trujillo R. D. Yue S. B. Tang Y. O’gorman W. E. Chen C. Z. (2010). The potential functions of primary microRNAs in target recognition and repression. *EMBO J.* 29 3272–3285. 10.1038/emboj.2010.208 20808284 PMC2957211

[B138] Uhm S. J. Gupta M. K. Yang J. H. Chung H. J. Min T. S. Lee H. T. (2010). Epidermal growth factor can be used in lieu of follicle-stimulating hormone for nuclear maturation of porcine oocytes in vitro. *Theriogenology* 73 1024–1036. 10.1016/j.theriogenology.2009.11.029 20106515

[B139] Unsal T. Konac E. Yesilkaya E. Yilmaz A. Bideci A. Ilke Onen H. (2009). Genetic polymorphisms of FSHR, CYP17, CYP1A1, CAPN10, INSR, SERPINE1 genes in adolescent girls with polycystic ovary syndrome. *J. Assist. Reprod. Genet.* 26 205–216. 10.1007/s10815-009-9308-8 19387820 PMC2682189

[B140] Ussar S. Griffin N. W. Bezy O. Fujisaka S. Vienberg S. Softic S. (2015). Interactions between gut microbiota, host genetics and diet modulate the predisposition to obesity and metabolic syndrome. *Cell Metab.* 22 516–530. 10.1016/j.cmet.2015.07.007 26299453 PMC4570502

[B141] Uzbekova S. Elis S. Teixeira-Gomes A. P. Desmarchais A. Maillard V. Labas V. (2015). MALDI mass spectrometry imaging of lipids and gene expression reveals differences in fatty acid metabolism between follicular compartments in porcine ovaries. *Biology (Basel)* 4 216–236. 10.3390/biology4010216 25756245 PMC4381227

[B142] Valckx S. D. De Pauw I. De Neubourg D. Inion I. Berth M. Fransen E. (2012). BMI-related metabolic composition of the follicular fluid of women undergoing assisted reproductive treatment and the consequences for oocyte and embryo quality. *Hum. Reprod.* 27 3531–3539. 10.1093/humrep/des350 23019302

[B143] Van Hoeck V. Leroy J. L. Arias Alvarez M. Rizos D. Gutierrez-Adan A. Schnorbusch K. (2013). Oocyte developmental failure in response to elevated nonesterified fatty acid concentrations: mechanistic insights. *Reproduction* 145 33–44. 10.1530/rep-12-0174 23108110

[B144] Van Hoeck V. Rizos D. Gutierrez-Adan A. Pintelon I. Jorssen E. Dufort I. (2015). Interaction between differential gene expression profile and phenotype in bovine blastocysts originating from oocytes exposed to elevated non-esterified fatty acid concentrations. *Reprod. Fertil. Dev.* 27 372–384. 10.1071/rd13263 24360349

[B145] Villa-Diaz L. G. Miyano T. (2004). Activation of p38 MAPK during porcine oocyte maturation. *Biol. Reprod.* 71 691–696. 10.1095/biolreprod.103.026310 15115730

[B146] Wang F. Tian X. Zhang L. Gao C. He C. Fu Y. (2014). Beneficial effects of melatonin on in vitro bovine embryonic development are mediated by melatonin receptor 1. *J. Pineal Res.* 56 333–342. 10.1111/jpi.12126 24666110

[B147] Wang F. Tian X. Zhang L. Tan D. Reiter R. J. Liu G. (2013). Melatonin promotes the in vitro development of pronuclear embryos and increases the efficiency of blastocyst implantation in murine. *J. Pineal Res.* 55 267–274. 10.1111/jpi.12069 23772689

[B148] Wang L. P. Peng X. Y. Lv X. Q. Liu L. Li X. L. He X. (2019). High throughput circRNAs sequencing profile of follicle fluid exosomes of polycystic ovary syndrome patients. *J. Cell. Physiol.* 18:28201. 10.1002/jcp.28201 30779115

[B149] Wang Z. B. Hao J. X. Meng T. G. Guo L. Dong M. Z. Fan L. H. (2017). Transfer of autologous mitochondria from adipose tissue-derived stem cells rescues oocyte quality and infertility in aged mice. *Aging* 9 2480–2488. 10.18632/aging.101332 29283885 PMC5764387

[B150] Wickham E. P. III Ewens K. G. Legro R. S. Dunaif A. Nestler J. E. Strauss J. F. III (2011). Polymorphisms in the SHBG gene influence serum SHBG levels in women with polycystic ovary syndrome. *J. Clin. Endocrinol. Metab.* 96 E719–E727. 21252242 10.1210/jc.2010-1842 PMC3070246

[B151] Wu L. L. Dunning K. R. Yang X. Russell D. L. Lane M. Norman R. J. (2010). High-fat diet causes lipotoxicity responses in cumulus-oocyte complexes and decreased fertilization rates. *Endocrinology* 151 5438–5445. 10.1210/en.2010-0551 20861227

[B152] Wu X. Q. Xu S. M. Liu J. F. Bi X. Y. Wu Y. X. Liu J. (2014). Association between FSHR polymorphisms and polycystic ovary syndrome among Chinese women in north China. *J. Assist. Reprod. Genet.* 31 371–377. 10.1007/s10815-013-0166-z 24390680 PMC3947065

[B153] Xiao S. Coppeta J. R. Rogers H. B. Isenberg B. C. Zhu J. Olalekan S. A. (2017). A microfluidic culture model of the human reproductive tract and 28-day menstrual cycle. *Nat. Commun.* 8:14584. 10.1038/ncomms14584 PMC5379057 28350383

[B154] Xu Y. Tan L. J. Grachtchouk V. Voorhees J. J. Fisher G. J. (2005). Receptor-type protein-tyrosine phosphatase-kappa regulates epidermal growth factor receptor function. *J. Biol. Chem.* 280 42694–42700. 10.1074/jbc.m507722200 16263724

[B155] Yamashita Y. Hishinuma M. Shimada M. (2009). Activation of PKA, p38 MAPK and ERK1/2 by gonadotropins in cumulus cells is critical for induction of EGF-like factor and TACE/ADAM17 gene expression during in vitro maturation of porcine COCs. *J. Ovarian Res.* 2:20. 10.1186/1757-2215-2-20 20034375 PMC2803446

[B156] Ye J. Li J. Yu Y. Wei Q. Deng W. Yu L. (2010). L-carnitine attenuates oxidant injury in HK-2 cells via ROS-mitochondria pathway. *Regul. Pept.* 161 58–66. 10.1016/j.regpep.2009.12.024 20093144

[B157] Zhang C. L. Wang H. Yan C. Y. Gao X. F. Ling X. J. (2017). Deregulation of RUNX2 by miR-320a deficiency impairs steroidogenesis in cumulus granulosa cells from polycystic ovary syndrome (PCOS) patients. *Biochem. Biophys. Res. Commun.* 482 1469–1476. 10.1016/j.bbrc.2016.12.059 27965096

[B158] Zhang J. Y. Jiang Y. Lin T. Kang J. W. Lee J. E. Jin D. I. (2015). Lysophosphatidic acid improves porcine oocyte maturation and embryo development in vitro. *Mol. Reprod. Dev.* 82 66–77. 10.1002/mrd.22447 25564987

[B159] Zhang L. Lu T. Wang Z. Meng L. Luo Y. Fu X. (2020). Mitochondrial Ca 2+ overload leads to mitochondrial oxidative stress and delayed meiotic resumption in mouse oocytes. *Front. Cell Dev. Biol.* 8:1053. 10.3389/fcell.2020.580876 PMC7770107 33384990

[B160] Zhao S. P. Tang X. M. Shao D. H. Dai H. Y. Dai S. Z. (2003). [Association study between a polymorphism of aldosterone synthetase gene and the pathogenesis of polycystic ovary syndrome]. *Zhonghua Fu Chan Ke Za Zhi* 38 94–97. 12783697

[B161] Zhao X. Jiang Y. Xi H. Chen L. Feng X. (2020). Exploration of the relationship between gut microbiota and polycystic ovary syndrome (PCOS): a review. *Geburtshilfe Frauenheilkd.* 80 161–171. 10.1055/a-1081-2036 32109968 PMC7035130

[B162] Zhu M. Shen Q. Li X. Kang J. (2020). Removal of peri-ovarian adipose tissue affects follicular development and lipid metabolismdagger. *Biol. Reprod*. 103 1199–1208. 10.1093/biolre/ioaa144 32813010

